# Genetics of migraine: where are we now?

**DOI:** 10.1186/s10194-023-01547-8

**Published:** 2023-02-20

**Authors:** Lou Grangeon, Kristin Sophie Lange, Marta Waliszewska-Prosół, Dilara Onan, Karol Marschollek, Wietse Wiels, Petr Mikulenka, Fatemeh Farham, Cédric Gollion, Anne Ducros, on behalf of the European Headache Federation School of Advanced Studies (EHF-SAS)

**Affiliations:** 1 Neurology Department, CHU de Rouen, Rouen, France; 2 Neurology Department, Charité – Universitätsmedizin Berlin, Berlin, Germany; 3 Center for Stroke Research Berlin (CSB), Charité – Universitätsmedizin, Berlin, Germany; 4 Department of Neurology, Wrocław Medical University, Wrocław, Poland; 5 Hacettepe University, Faculty of Physical Therapy and Rehabilitation, Ankara, Turkey; 6 Department of Neurology, Universitair Ziekenhuis Brussel, Vrije Universiteit Brussel, Brussels, Belgium; 7 Department of Neurology, Third Faculty of Medicine, Charles University and University Hospital Kralovske Vinohrady, Prague, Czech Republic; 8 Headache Department, Iranian Centre of Neurological Researchers, Neuroscience Institute, Tehran University of Medical Sciences, Tehran, Iran; 9 Neurology Department, CHU de Toulouse, Toulouse, France; 10 Neurology Department, CHU de Montpellier, 80 avenue Augustin Fliche, 34295 Montpellier, France

**Keywords:** Migraine, Familial hemiplegic migraine, Genetics, Genome-wide association studies, Polygenic

## Abstract

Migraine is a complex brain disorder explained by the interaction of genetic and environmental factors. In monogenic migraines, including familial hemiplegic migraine and migraine with aura associated with hereditary small-vessel disorders, the identified genes code for proteins expressed in neurons, glial cells, or vessels, all of which increase susceptibility to cortical spreading depression. The study of monogenic migraines has shown that the neurovascular unit plays a prominent role in migraine. Genome-wide association studies have identified numerous susceptibility variants that each result in only a small increase in overall migraine risk. The more than 180 known variants belong to several complex networks of “pro-migraine” molecular abnormalities, which are mainly neuronal or vascular. Genetics has also highlighted the importance of shared genetic factors between migraine and its major co-morbidities, including depression and high blood pressure. Further studies are still needed to map all of the susceptibility loci for migraine and then to understand how these genomic variants lead to migraine cell phenotypes.

## Migraine, a complex genetic condition

The goal of genetics is to identify key proteins in order to better understand the pathophysiology of a disease, to define new therapeutic targets and to find diagnostic biomarkers. Migraine is a highly disabling, complex brain disorder with a strong familial aggregation. Twin and family studies conducted in the 1990s demonstrated the existence of hereditary factors in migraine [1, 2]. In these studies, the estimated heritability of migraine ranged from 35% to 60%. In population-based studies, the relative risk of migraine for a first-degree relative of an index case was 1.5- to 4-fold compared with the general population [3]. The risk was higher for relatives of cases with higher pain scores and attack frequency, early age of onset, and migraine with aura (MwA).

More recent studies estimate the heritability of migraine to be about 42%. They also reinforce the idea that migraine is a complex disease resulting from interactions between genes and the environment, interactions between genes themselves, and as yet unknown factors [3]. Heritability is higher in MwA than in migraine without aura (MO) [4].

**Fig. 1:**
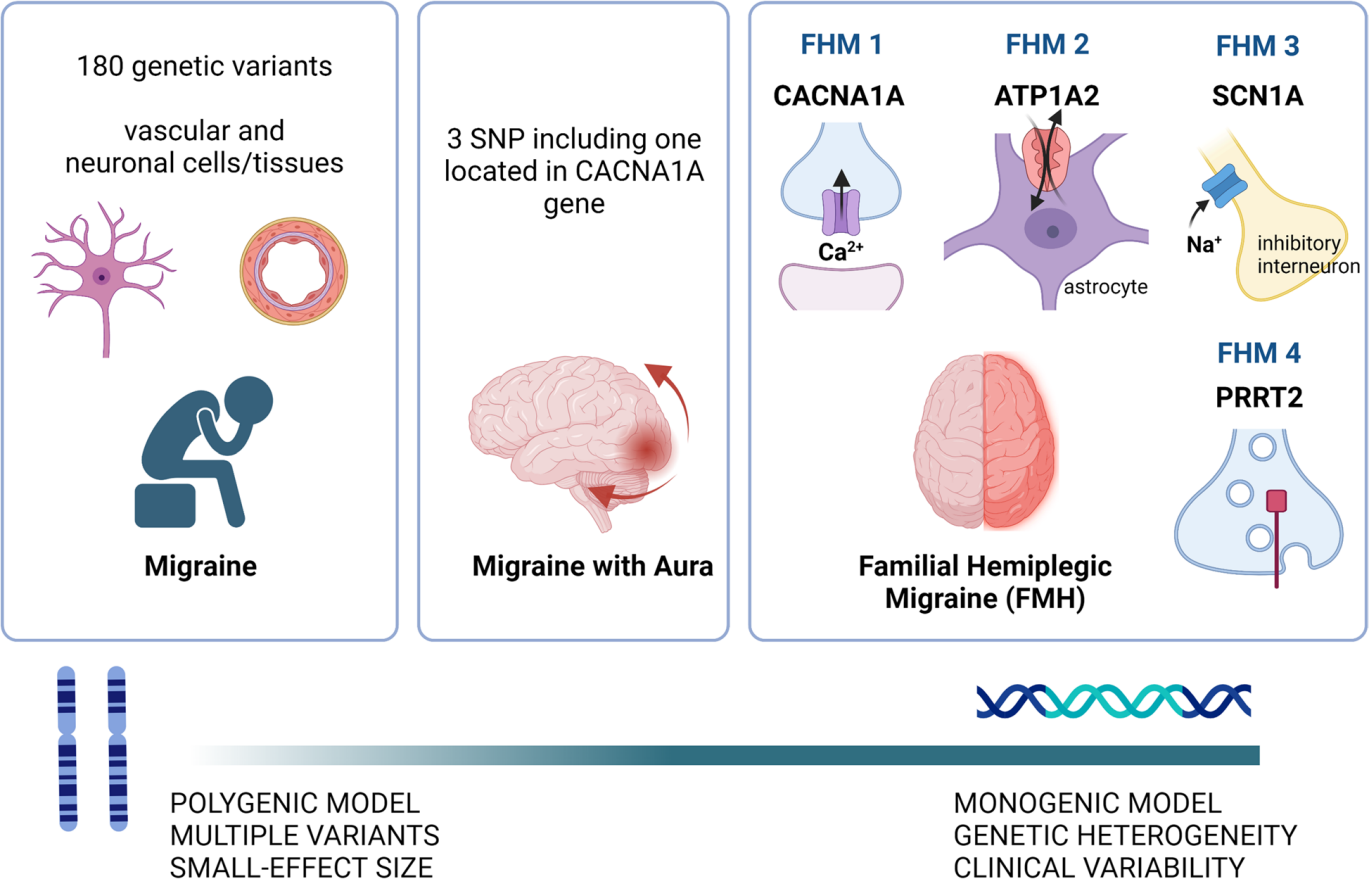
Overview of migraine and genetics. An overview of the complex genetic architecture of migraine, from polygenic model on the left, to monogenic model on the right. *Created with BioRender.com*

Migraine is predominantly polygenic, with multiple genetic variants, each with a minor-effect size, accumulating to lead to the disease. A portion of MwA cases could be explained by the conjunction of a small number of genetic variants with moderate effect size, or by a single variant with a major functional effect as in monogenic migraines [5] (Fig. 1). In these much rarer disorders, a pathogenic mutation in a single gene is sufficient to produce the disease with almost complete penetrance. The classical example of monogenic migraine is familial hemiplegic migraine (FHM), which is inherited in an autosomal dominant fashion [6]. Migraines can also be part of the clinical spectrum of other hereditary neurological conditions, such as cerebral autosomal dominant arteriopathy with subcortical infarcts and leukoencephalopathy (CADASIL). Other examples will be discussed further in this paper.

## Familial hemiplegic migraine (FHM), a monogenic form of migraine

### Genetic heterogeneity and clinical variability

Hemiplegic migraine (HM) is a rare disease with an estimated prevalence of 0.01% in the general population [6, 7]. Familial HM, diagnosed when at least one first- or second-degree relative also has HM, accounts for two-third of the cases. Sporadic HM (SHM), diagnosed in the absence of family history, accounts for one-third of cases. HM attacks begin during youth (mean age of onset 12–17 years old), and comprise motor weakness during the aura, always associated with at least one other symptom of typical aura (visual, sensory, speech and language) and often with brainstem aura symptoms (70%) [8, 9]. The frequency of attacks varies from more than one per week to a few over the course of a lifetime, with an average of 3 to 4 per year [10]. Duration of HM aura is often longer than that of typical aura (several days to weeks) [11–13]. Severe attacks with confusion, coma, fever, seizures and reversible brain edema may occur [14–17], sometimes triggered by mild head trauma [18–20]. HM can be pure or associated with a combination of early-onset epilepsy, cerebellar ataxia, learning disabilities, and/or mental retardation, which may begin before or after HM onset [21–25].

**Fig. 2:**
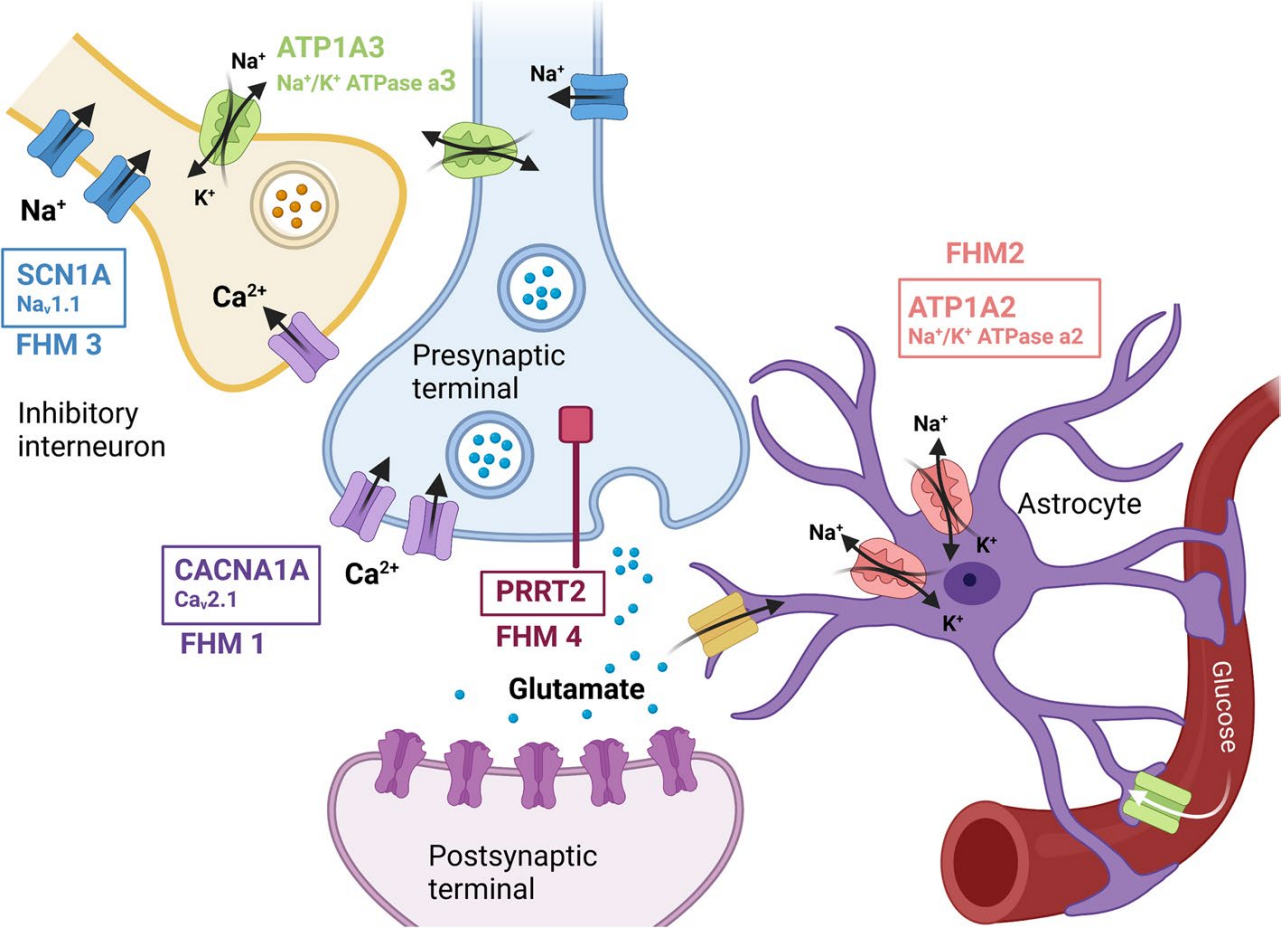
Genetics of familial hemiplegic migraine (FHM). Glutamatergic synapse of the central nervous system with proteins encoded by genes involved in familial hemiplegic migraine and their functional roles. *Created with BioRender.com*

FHM is primarily a monogenic disorder, with an autosomal dominant pattern of inheritance and high penetrance; 70–90% of individuals with a pathogenic mutation clinically express the disease. FHM is genetically heterogeneous and is subdivided into FHM1, FHM2 and FHM3, based on the presence of mutations in the *CACNA1A*, *ATP1A2* and *SCN1A* genes, respectively [6, 26–28] (Table 1, Fig. 2). The *PRRT2* gene should be added to the main FHM genes because recent data have shown that *PRRT2* is involved at least as frequently as *SCN1A* [29, 30]. For convenience, we will therefore refer to FHM4 for HM associated with *PRRT2* mutations. Other genes have been reported in a small number of cases and families, and additional data are needed before they can be considered causal (Table 2).

**Table 1:** The four major genes implicated in familial hemiplegic migraine

Genes	Approximate number of cases and families reported in the literature^a^		Percentage of affected subjects in four cohorts: a. Sutherland et al, 2020 [29] b. Pelzer et al, 2018 [21] c. Riant et al 2022 [30] d. Hiekkala et al, 2018 [31]	Nature of causative mutations	Protein encoded and role	Involvement of the gene in other conditions [32]
***FHM1: CACNA1A***	> 600 cases	150 families	a. 16/203 (7%) b. 107/? c. 26/697 (3.7%) d. 1/293 (0.34%)	Missense mutations (Gain of function) Rare large exonic deletion or deletion in 5′ non coding end promoter	Alpha-1 subunit of neuronal Ca_v_2.1 (P/Q type) voltage-gated calcium channels ➔ Control of neuronal excitability at the presynaptic level of glutamatergic synapsis	Episodic ataxia type 2 (loss of function) Spinocerebellar ataxia type 6 (SCA6) Lennox-Gastaut syndrome or Dravet syndrome, autism spectrum disorder (gain and loss of function) Possibly involved in Benign paroxysmal positional vertigo Dyskinesia Rett syndrome Paroxysmal tonic upward gaze
***FHM2: ATP1A2***	> 900 cases	160 families	a. 20/203 (10%) b. 75/? c. 44/697 (6.3%) d. 2/293 (0.68%)	Missense mutations Rare small deletions or truncating mutations, frameshift	Catalytic alpha-2 subunit of the glial and neuronal ATP-dependent trans membrane Na^+^/K^+^-pump ➔ Clearance of extracellular K^+^ and production of a Na^+^ gradient necessary for glutamate reuptake	Alternating hemiplegia of childhood Rapid-onset dystonia parkinsonim Cerebellar ataxia-areflexia-progressive optic atrophy Severe childhood epilepsies, encephalopathy and polymicrogyria Mental retardation
***FHM3: SCN1A***	> 120 cases	20 families	a. 4/203 (1.7%) b. 26/? c. 15/697 (2.1%) d. 0/293	Missense mutations (gain of function)	Alpha-1 subunit of neuronal Na_v_1.1 voltage-gated sodium channels ➔ Propagation of action potentials of cortical neurons, especially in GABAergic inhibitory interneurons	Early onset epileptic encephalopathies (cryptogenic generalized epilepsy, cryptogenic focal epilepsy, myoclonic–astatic epilepsy, Lennox-Gastaut syndrome, infantile spasm, Rasmussen encephalitis
***FMH4: PRRT2***	> 120 cases	40 families	a. 5/203 (2.2%) b. 1/47 (2.1%) c. 30/697 (3.5%) d. not screened	Missense mutations	Pre-synaptic proline-rich transmembrane protein ➔ Interaction with the synaptosomal-associated protein 25 (SNAP25), suggesting a role in the fusion of synaptic vesicles to the plasma membrane	Paroxysmal kinesigenic dyskinesia Benign familial infantile epilepsy Infantile convulsions with choreoathetosis Episodic ataxia Paroxysmal torticollis Intellectual disability

^a^Based on research on Pubmed using keywords “name of the gene (example: *CACNA1A*)” and “migraine” or “name of the gene” and “headache”. Articles in other language than English, focusing on mice studies or other conditions than migraine were excluded. Each case where family history was not detailed was considered as sporadic and did not count as one family

**Table 2:** Other genes potentially implicated in familial hemiplegic migraine (FHM)

Genes potentially implicated	Number of cases and families reported in literature		Percentage of affected subjects in the cohort by Sutherland et al, 2020 [29]	Protein encoded and role	Involvement of the gene in other conditions	References
***PKND***	4	2	0,4%	PKND protein ➔ Interaction with proteins of the synaptic termini to modulate the release of neurotransmitters	Main gene for paroxysmal non-kinesigenic dyskinesia	[33, 34]
***SLC4A4***	15	11	3,9%	Na + −HCO3 NBCe1 cotransporter ➔ Expressed in astrocytes, regulation of synaptic pH and neurotransmission	Renal tubular acidosis	[35–37]
***ATP1A3***	3	3	0,9%	α3 subunit of the Na+/K + -ATPase pump ➔ Maintain of electrochemical gradients across neuronal membranes and regulation of excitability at inhibitory synapses	Alternating hemiplegia of childhood	[38, 39]
***SLC1A3***	4	4	0,4%	EAAT1 transporter ➔ Capture of glutamate into astrocytes	Episodic ataxia type 6	[40–43]
***SLC2A1***	6	6	1,3%	Glucose transporter GLUT1 (or EAAT2) ➔ Entry of glucose into the brain across the blood-brain barrier	Paroxysmal exercise-induced dyskinesia, De Vivo disease and GLUT1 deficiency syndrome	[33, 44–47]
***ATP1A4***	15	13	5.2%	Sodium/potassium-transporting ATPase subunit alpha-4	Charcot-Marie-Tooth Disease, Axonal, Type 2Dd Alternating hemiplegia of childhood	[29, 48]

Clinical studies of patients with mutations in the four major FHM genes have shown that attacks of HM are similar regardless of the gene involved, and that prolonged auras with confusion are possible in all FHM types. The association of HM with epilepsy is present in 7% of overall HM patients [7, 49], but seems more frequent in FHM2 [50].

Conversely, different mutations in the same gene can influence the phenotype. In FHM1, the two mutations most commonly involved in severe attacks with coma and fever are T666M and S218L [17]. Moreover, the nature of the mutated gene also influences the spectrum of manifestations associated with HM attacks [6]. Febrile comas are frequent in FHM1 (up to 30%), possible in FHM2 (up to 15%), and have not been described in FHM3 and FHM4 [17, 51]. Cerebellar ataxia is common in FHM1 [52–56]; a phenomenon of repeated transient blindness was observed only in FHM3 [57]; mental retardation has been described in FHM1, 2 and 4 [22–24]; and finally, the association of HM with paroxysmal dyskinesia or hypersomnia is suggestive of FHM4 [30]. Finally, there is great variability in HM attacks and associated manifestations between individuals who carry different mutations in the same gene, and even between affected family members who carry the same mutation. This variability suggests that other genetic or environmental factors can modulate the clinical phenotype [26].

### FHM1 and CACNA1A mutations

*CACNA1A*, localized on 19p13, was the first identified HM gene [58]. It encodes the main α1 pore-forming subunit of the neuronal voltage-gated calcium channels Ca_V_2.1 or P/Q. These channels are expressed in synaptic endings in the brain and the cerebellar, and play a role in controlling neurotransmitter release. More than 25 *CACNA1A* mutations have been identified in FHM1. Most are missense mutations resulting in a gain of function, which increases Ca^2+^ influx, glutamatergic neurotransmission and neuronal excitability [59].

There are two transgenic FHM1 knock-in (KI) mouse models [60, 61]. KI mice for the R192Q mutation, which causes pure FHM1, show no clinical abnormalities. KI mice for the S218L mutation, which causes very severe FHM1, show cerebellar ataxia, transient hemiparesis and epilepsy. FHM1-KI mice exhibit increased Ca_V_2.1 currents and neurotransmitter release, loss of balance between excitatory and inhibitory cortical neurotransmissions, enhanced cortical excitatory transmission in visual cortex [62] and increased susceptibility to cortical spreading depression (CSD). These transgenic mice have also been shown to exhibit head pain [63], increased trigeminal activity, tissue anoxia during prolonged aura, increased sensitivity to cerebral ischemia, and altered trigeminal nociception mediated by CGRP [64].

The *CACNA1A* gene is also mutated in other neurological disorders. Episodic ataxia type 2 (EA2), characterized by paroxysmal ataxia, dizziness and nausea, is associated with *CACNA1A* mutations responsible for loss of function and decreased Ca^2+^ influx [65]. Spinocerebellar ataxia type 6 (SCA6), characterized by progressive cerebellar ataxia, is caused by an expansion of a CAG repeat in the terminal portion of *CACNA1A*, which results in toxic degeneration of cerebellar Purkinje cells [16].

### FHM2 and ATP1A2 mutations

The *ATP1A2* gene on 1q23.2 encodes the α2 isoform of the catalytic subunit of the A1A2 ATP-dependent transmembrane pump (α_2_ Na^+^/K^+^-ATPase) [66]. In the CNS of adults, this pump is primarily expressed in astrocytes, where it provides extracellular K^+^ clearance and produces a Na^+^ gradient necessary for glutamate reuptake from the synaptic cleft. More than 80 *ATP1A2* mutations have been identified in FHM2. Missense mutations are the most common, but small deletions, a stop-codon altering mutation, and an exonic duplication have also been reported. These mutations result in a variable loss of function of the α_2_ Na^+^/K^+^-ATPase pump. The mutated pumps are reported to have lower glutamate uptake, slowing down recovery from neuronal excitation and promoting excitatory cortical transmission, thereby facilitating the initiation of CSD waves. There are several models of FHM2-KI transgenic mice. Heterozygous transgenic mice show no clinical abnormalities but have increased susceptibility to CSD [67, 68]. Mice with partially knock-out (KO) of *ATP1A2* also show increased susceptibility to CSD [69]. Another mouse model with complete KO of *ATP1A2* in astrocytes showed episodic paralysis and spontaneous waves of CSD with decreased EEG activity [70]. These animals had abnormalities in brain metabolism with increased serine and glycine. A serine- and glycine-free diet suppressed attacks of paralysis in these mutants.

### FHM3 and SCN1A mutations

*SCN1A* on 2q24.3 encodes the α_1_ subunit forming the pore of Na_V_1.1 channels [71]. These voltage-dependent neuronal sodium channels are involved in the genesis and propagation of action potentials in cortical neurons, particularly in GABAergic inhibitory interneurons [72]. *SCN1A* was already known as an epilepsy gene with over 100 missense and nonsense mutations identified in various forms of childhood epilepsies. A dozen *SCN1A* mutations, mainly missense variants leading to a gain of function, have been identified in FHM3. Their functional consequences are complex [73, 74]. The mouse model carrying the L1649Q variant showed an increased susceptibility to CSD. The L1649Q mutation results in a defect in Na + channel inactivation with increased Na + currents and hyperactivity of inhibitory interneurons.

### FHM4 and PRRT2 mutations

*PRRT2* encodes the PRRT2 protein, which plays an important role in brain development, synapse formation, and neurotransmitter release. PRRT2 is expressed in presynaptic terminals and interacts with proteins of the exocytosis complex. Mutations in *PRRT2* have now been identified in several dozen cases of FHM4, two-thirds of which have pure FHM, and one-third of which have FHM associated with epilepsy, mental retardation or dyskinesia [21, 75–80]. Mutations in *PRRT2* are also associated with several other neurological diseases, including benign familial infantile epilepsy (BFIE), infantile seizure syndrome with choreoathetosis (ICCA) and paroxysmal kinesigenic dyskinesia (PKD) [81, 82].

The different mutations in *PRRT2* (point duplication, small deletions, missense, total deletions) all induce a loss of function leading to haploinsufficiency. A given *PRRT2* mutation can be associated with several diseases. Indeed, the c.649dupC mutation is common in FHM4, but is also the main causative mutation in PKD and BFIE.

PRRT2-KO mice exhibit paroxysmal abnormal movements upon acquisition of locomotion, develop abnormal audiogenic motor behaviors in adulthood, and have a lowered seizure threshold [83]. Their excitatory hippocampal neurons display increased excitability. Human and murine homozygous KO-PRRT2 neurons in culture express overactive Na_V_1.2 and Na_V_1.6 channels, indicating that PRRT2 inhibits voltage-gated sodium channels.

Further experiments are needed to understand the factors underlying the great phenotypical variability associated with *PRRT2* mutations, and the potential influence of modifier genes or of the non-mutated allele.

### Other potential FHM genes

Mutations in several other genes have been identified in HM (Table 2) [29]. All these genes were already known to be involved in other inherited diseases. In a large cohort of index HM cases from New-Zealand and Australia, analysis of potential new HM genes increased the diagnosis rate from 21% to 27,8% (*PKND* 0,4%; *SLC4A4* 3,9%; *ATP1A3* 0,9%; *SLC1A3* 0,4%; *SLC2A1* 1,3%) [29]. Analysis of other large cohorts of index cases, as well as functional studies assessing CSD in animal models would be important to confirm that these genes actually cause FHM.

### Genetic architecture of hemiplegic migraine

Among index cases suspected of having HM and referred for genetic diagnosis, a minority has a mutation in one of the four major genes, 15% in a French cohort of 697 patients [78], and 21% in a New Zealand and Australian cohort of 230 patients [29]. These two independent studies yielded similar results, with the most frequent mutations found in the *ATP1A2* gene (6,3–10%) followed by *CACNA1A* (3,7–7%), *PRRT2* (2,2-3,5%) and finally, *SCN1A* (1,7 -2,1%). In contrast, a Dutch study of a cohort of 301 patients found higher rates of mutations in major genes: *CACNA1A* in 107/301 (35.5%), *ATP1A2* in 75/301 (24.9%), *SCN1A* in 26/301 (8.6%), and *PRRT2* in 1 /47 (2.1%) [21]. In addition, only three mutations were identified in a Finnish cohort of 293 HM patients: one in *CACNA1A* (0.34%) and two in *ATP1A* 2(0.68%) [31]. The *PRRT2* gene was not screened in the Finnish study [31]. These differences could be due to different recruitment methods of the cohorts.

In typical HM cases in whom there are no mutations in the four main genes, additional single-gene variants may be identified by future systematic studies, such as exome studies, and full genome sequencing. New variants with large-effect sizes are expected to be involved in only a small proportion of familial and sporadic cases. In other cases of HM, the inheritance may be polygenic, involving multiple variants with each a small-effect size, or oligogenic, with a combination of one or few variants with a moderate-effect size with or without multiple pro-migraine variants of small-effect size.

SHM, diagnosed in the absence of any affected relative, can result from a de novo mutation of one of the FHM genes in a subject whose two parents do not have a mutation [47, 84–88]. These de novo mutations can be passed to offspring, transforming SHM into FHM. SHM can also result from mutations in known FHM genes with low penetrance, mosaicism in the transmitting parent, or pathogenic variants in as yet unknown genes. Other SHM types might have a different mode of inheritance, either recessive with compound heterozygotes or polygenic [26]. Finally, environmental and psychosocial factors including exposure to stress, psychological and physical trauma, abuse, or negative life events may also play an important role in SHM. According to the US military personnel HM cohort, the incidence of HM was zero from 1997 to 2007, and then steadily increased, with a 25-fold increase in new cases between 2008 and 2017 [89].

### Links between FHM and the common varieties of migraine

FHM is a model of hereditary severe migraine with aura. Some authors have suggested that the mechanisms of FHM, namely increased sensitivity to CSD, may be involved in common migraine, with and without aura. Danish studies have shown that the risk of migraine with typical aura (eg without motor deficit) was increased in individuals with FHM compared with the general population, whereas the risk of MO was similar [90, 91]. Thus, FHM is a major model for migraine aura associated with cortical excitability, with subsequent headache triggered by CSD, and FHM genes do not play a major role in the genetics of the common varieties of migraine [92, 93]. A recent study has identified a polymorphism in the FHM1 *CACNA1A* gene as a susceptibility locus for common varieties of migraine, among 122 other loci [94].

## Other monogenic varieties of migraine

### Monogenic migraine with aura and TRESK mutations

TRESK is a two-pore K^+^ channel responsible for maintaining membrane excitability. By a free flow of K^+^ ions, it contributes to the formation of leakage currents in the trigeminal ganglion and dorsal root ganglia. It is therefore though to play a role in pain processing mechanisms. Mice with a functional knock-out of TRESK show a ‘painful’ behavioural phenotype, and exhibit hyperexcitability of the dorsal root and trigeminal ganglia. In addition, in the trigeminal ganglion, TRESK expression is restricted to nociceptive neurons [5, 95].

A frameshift mutation in *KCNK18*, which encodes the TRESK channel, was described in a large family with visual MwA following autosomal dominant inheritance [96]. All family members with migraine carried the p.(F139Wfs*24) mutation, which has been shown to exert a dominant negative effect resulting in complete loss of TRESK function and increased neuronal excitability [97].

The causal link between TRESK mutations and migraine has been called into question by the discovery of another mutation with a dominant negative effect, C110R, in individuals without migraine [98]. However, further research revealed that the p.(F139Wfs*24) variant introduces an alternative start codon that shortens the TRESK protein and damages its function resulting in hyperexcitability of nociceptors. Such effects were not observable for C110R [97, 99]. Another missense mutation in the KCNK18 gene (W101R) was identified in a 12-year-old male with migraine with brainstem aura and intellectual disability. This variant was inherited from his mother, who had migraine with aura [100]. Further investigations revealed impaired TRESK channel function associated with this variant [101]. In another study, pharmacological inhibition of TRESK influenced the response to capsaicin, a TRPV1 receptor agonist, and resulted in increased CGRP release and meningeal blood flow [102]. These data suggested that MwA can be caused by a rare genetic variant inducing a major functional effect.

A study of 200,000 exome-sequenced UK Biobank participants conducted in 2022 found the frameshift variant p.(F139Wfs*24) in 196 (0.10%) of the 193,433 participants classified as controls and in 10 (0.14%) of the 7194 migraine cases (*p* = 0.33) [103]. The authors concluded that *KCNK18* should no longer be regarded as being involved in migraine etiology. A major limitation of this study is that the clinical status of healthy controls was not assessed in details. This may imply that a proportion of the 193,433 participants may in fact have been affected by migraine, as mis- and underdiagnosis of migraine and migraine aura is highly common [104]. Studies of cohorts of patients with a firm diagnosis of MwA and well characterized healthy controls are needed to further elucidate the role of *KCNK18* and its product, TRESK in migraine.

### Familial advanced sleep-phase syndrome (FASP), migraine and *CSNK1D* mutations

FASPS, which causes an extreme tendency to wake-up early in the morning, can be caused by mutation in a circadian clock gene, *CSNK1D*, which codes for casein kinase 1 delta (CKIδ). In the two large families with a CKIδ mutation, the sleep disorder was associated with migraine [105]. Transgenic mice expressing T44A variant of *CSNK1D* displayed a high propensity for nitroglycerin-induced mechanical hyperalgesia, and a reduced threshold for CSD. These findings suggest that migraine may be caused by a mutation in a gene that encodes neither an ion channel nor a protein involved in glutamate signaling. In addition, the link between migraine and FASPS is consistent with the known role of hypothalamus in migraine [106, 107].

### ROSAH syndrome, migraine and *ALPK1* mutations

ROSAH syndrome (retinal dystrophy, optic nerve edema, splenomegaly, anhidrosis, migraine headache) is an autosomal dominant condition caused by a missense mutation in the *ALPK1* gene which was identified in five families. *ALPK1* encodes Alpha Kinase 1, which plays a role in inflammation, cellular trafficking, and possibly also affects CGRP activity [26, 108]. Its role in migraine is unknown.

### Monogenic cerebral vasculopathies and migraine

CADASIL (Cerebral autosomal dominant arteriopathy with subcortical infarcts and leukoencephalopathy) is the commonest type of familial cerebral small-vessel disease responsible for recurrent lacunar stroke, leading to dementia and premature death [27, 109]. CADASIL is caused by *NOTCH3* mutations, which cause progressive destruction of vascular smooth muscle cells [110]. Migraine is very common in CADASIL (up to 75%), mainly migraine with typical aura, but also hemiplegic migraine or migraine without aura. Migraine is often the first manifestation of the disease, 15 to 20 years before cerebral infarctions [111, 112]. Mutant mice, either knock-in for a CADASIL mutation or *NOTCH3*-knock-out, have shown increased sensitivity to CSD [113].

Retinal vasculopathy with cerebral leukodystrophy (RVCL) caused by mutations in *TREX1*, and disorders due to *COL4A1* and *COL4A2* mutations are other small-vessel diseases that frequently involve migraines [27].

The study of these conditions, especially CADASIL, showed that gene expressed only in vessels could be implicated in migraine, which was later on confirmed by studies in polygenic migraines.

## Susceptibility genes for migraine with aura and migraine with aura

### Genome-wide association studies (GWAS)

Identification of gene variants involved in migraine has proven difficult, and there have been 30 years of studies without significant results. Because MO and MwA display strong familial aggregation, which may suggest Mendelian inheritance, the initial hope was that the techniques used successfully in FHM would identify the genes for the most common migraines. Initial studies showed that the FHM genes were not involved in the common varieties of MO and MwA. Linkage analysis studies identified dozens of loci that were presented as possibly containing genes involved in migraine, but these were never discovered [5, 114].

Researchers turned to genome-wide association study (GWAS), which examines millions of polymorphisms called SNPs (single nucleotide polymorphisms) in very large cohorts of patients and healthy controls. Each SNP is a variation in the genetic code at a single DNA base pair. More than 100 million SNPs exist in the human genome, and 4–5 million SNPs are distributed throughout an individual genome [115]. A GWAS identifies SNPs that are significantly associated with the disease of interest, by assessing differences in allele frequencies between large numbers of patients and controls. For each SNP, the level of significance is very difficult to reach (5 × 10^− 8^) because of the multiplicity of tests performed.

In 2010, the first GWAS identified a single migraine susceptibility locus [116] (Table 3) Over the past decade, the International Headache Genetics Consortium (IHGC; www.headachegenetics.org/) has conducted several migraine GWAS, and with increasing sample sizes, the number of associated genetic variants has progressively increased [117–119]. The 2016 migraine GWAS, including 59,674 migraine sufferers and 316,078 controls, identified 38 distinct genomic loci associated with migraine [120]. Tissue expression enrichment analyzes clearly demonstrated the enrichment in genes involved in arterial and smooth-muscle function [120, 121]. The other pathways identified were the neuronal pathway [122], and the pathway related to homeostasis of iron ions and other metals [120].

**Table 3:** Genome wide association studies in migraine from 2010 to 2022

Article	Population						Number of SNPs tested	Number of loci identified, ***P*** < 5 × 10^**−8**^	Number of new loci, ***P*** < 5 × 10^**− 8**^	Genes or nearest genes
	Origin	Migraine (n)	MO (n)	MwA + MO (n)	MwA only (n)	Controls (n)				
Antilla et al., 2010 [116]	Europeans	2748	17	2142	589	10,747	429,912	1 *12*^a^	1 *12*^a^	Intergenic between MTDH/AEG-1 and PGCP *MTDH/AEG-1 PGCP, SMYD3, INSIG2, TRPM8, MYLK4, ZNF311, NAV2, COG3, SGCZ, AQP9*.^a^
Ligthart et al., 2011 [123]	Europeans	2446	NA	NA	NA	8534	2,394,913	0 *10*^a^	0 *10*^a^	- *NGFR, AGBL1, MACC1, LIPG, AGA, KIF20B, BMP2, IGLL1, TSPAN2, KDM4C.*^a^
Chasman et al., 2012 [117]	American women with European ancestry	5122	1826	1177		18,108	2,608,509	0 *7*^b^	0 *7*^b^	- *PRDM16, TRPM8, SEPT7, C8orf79, LRP1*.^b^
Freilinger et al., 2012 [124]	Europeans	2326	2326	0	0	4580	Approximately 1.4 M	1 *12*^a^	1 *12*^a^	MEF2D *MEF2D, HFM1, RABGAP1L, MARCH4, TGFBR2, RGS12, PHACTR1, FHL5, ADAM28, ASTN2, INA, MMP17*.^a^
Anttila et al., 2013 [118]	European descent	23,285	7107	5118		95,425	Approximately 2.3 M	12	5	AJAP1, TSPAN2, FHL5, c7orf10, MMP16, PRDM16, MEF2D, TRPM8, TGFBR2, PHACTR1, ASTN2, LRP1.
Gormley et al., 2016 [120]	European descent	59,674	8348	6332		316,078	8,045,569	38	28	LRP1/STAT6/SDR9C7, PRDM16, FHL5/UFL1, TSPAN2/NGF, TRPM8/HJURP, PHACTR1, MEF2D, SLC24A3, FGF6, C7orf10, PLCE1, KCNK5, ASTN2, MRVI1, HPSE2, CFDP1, RNF213, NRP1, SPR149, JAG1, REST/SPINK2, ZCCHC14, HEY2/NCOA7, WSCD1/NLRP1, GJA1, TGFBR2, ITPK1, ADAMTSL4/ECM1, CCM2L/HCK, YAP1, MED14/USP9X, DOCK4/IMMP2L, CARF, ARMS2/HTRA1, IGSF9B, MPPED2, NOTCH4.
Chen et al., 2017 [125]	Asians (Han Chinese)	1005	1005	0	0	1053	642,832	0 *4*^c^	0 *4*^c^	- *DLG2, GFRA1, UPP2, GPR39.*^c^
Chang et al., 2018 [126]	African-Americans	380	123	40		2129	522,471	1	1	Near NMUR2 and GLRA1
Choquet et al., 2021 [127]	Multiethnic (East Asian, African American, Hispanic/Latino, European descent)	88,526	NA	NA	NA	841,795	Over 10 M	79	45	JAG1, LEPR, REST, HEY2, NGF, TGFB1, HOXB3, LRP1, PLCE1, HOXB2, HOXB6, YAP1, POLR2A, AMBRA1, TJP2, EP300, LRP4, ARHGAP1, ECM1, HOXB5, HOXB1, MRVI1, ZC3H7B, HTRA1, RPRD2, ZCCHC14, STAT6, MAPKAPK2, C7orf10, KCNK5, C20orf112, ZBTB4, POU4F1, C14orf37, IQCK, C1orf51, LINC00472, BTBD16, MPPED2, YRDC, PRPF3, ARID4A, GPRC5B, THADA, ZNF408, TMEM51, CHRNB1, CHST6, MTF1, MEF2D, INPP5B, CKAP5, RNF213, DGKZ, TSPAN2, CFDP1, KIAA0586, FHL5, ATG13, TIMM9, F2, PSMA3, GPR26, PLEKHA1, TMEM170A, PHB, SLC24A3, L3MBTL2, TARS2, DYRK3, MRPS21, FXN, CHRM4, TMEM91, CHADL, MANEAL, HARBI1, RANGAP1, IER3IP1, UFL1, C1orf122, TRPM8, PHACTR1, ACTR10, NCOA7, C11orf49, PRDM16, SLC45A1-RERE, TMEM51, TSPAN2-NGF, MSL3P1-TRPM8, MIR4791-EFHB, LINC00472-RIMS1, GJA1-HSF2, FXN, ASTN2-LOC100128505, GATA3-SFTA1P, MRGPRE-ZNF195, CALCB, FGF23-FGF6, LRP1-MIR1228, B3GNTL1-METRNL, LINC00310-KCNE2.
Hautakangas et al., 2022 [94]	Europeans	102,084	15,055	14,624		771,257	10,843,197	123	86	PRDM16, CAMTA1, TMEM51, INPP5B, MACF1, C1orf87, LEPR, RP4-598G3.1, TGFBR3, TSPAN2, ADAMTSL4, MEF2D, RABGAP1L, PLA2G4A, MAPKAPK2, KIF26B, THADA, ANKRD36C, ZEB2, AC064865.1, RNU6-546P, MYO3B, HOXD10, CARF, TRPM8, TGFBR2, ATRIP, HNRNPA3P8, CADM2, C3orf38, ITGB5, GPR149, SEC63P2, SPINK2, ANKDD1B, SSBP2, ZNF474, SNX24, POU4F3, TIGD6|HMGXB3, NKX2–5, NSD1, PHACTR1, PRL, IER3, EHMT2, KCNK5, KRT19P1, FHL5, REV3L, GJA1, PCMT1, SUGCT, MLXIPL, TSPAN12, PTK2B, RP11-573 J24.1, NFIB, RP11-373A6.1, TJP2, ZNF462, ASTN2, EHMT1, RNA5SP299, MLLT10, PLCE1, HPSE2, CNNM2, RBM20, HTRA1, GPR26, INPP5A, MRGPRE, MRVI1, INSC, MPPED2, AMBRA1, RAB3IL1, RBM14-RBM4|RBM4, YAP1, SPATA19, FGF6, PDZRN4, LRP1, ATP2B1, RP11-690 J15.1, NCOR2, LRCH1, RNF219-AS1, COL4A1, RP11-384 J4.2, LRFN5, ARID4A, DLST, IFT43, ITPK1, SERPINA1, ABHD17C, HMOX2, CFDP1, ZCCHC14, SMG6, ZBTB4, HOXB3, RP11-81 K2.1, MRC2, TBC1D16, RNF213, RBBP8, SKOR2, FECH, CACNA1A, SUGP1, B9D2|TMEM91, JAG1, SLC24A3, C20orf112, ZMYND8, MRPS6, RUNX1, AC006547.14, FAM47A, MED14

*MO* Migraine without aura, *MwA* Migraine with Aura, *M* Millions, *NA* Non Applicable, *SNPs* Single Nucleotide Polymorphisms

^a^Significant association with *p*-values ≤5 × 10^− 5^

^b^Significant association with *p*-values ≤5 × 10^− 6^

^c^Significant association with *p*-values ≤5 × 10^− 4^

In a third recent migraine GWAS from 2021, 79 independent loci were significantly associated with migraine [127]. Of note, this was an ethnically diverse study that included adult individuals (28,852 cases vs. 525,717 controls) from East Asian, African American, and Hispanic/Latino descent.

The most recent migraine GWAS published in 2022 by Hautakangas et al. [94] included 102,084 cases and 771,257 controls and identified 123 distinct loci associated with migraine, of which 86 were novel compared to the 2016 GWAS. Additional analyses even increased the number of independent SNPs to 167. Enrichment analyses in the 2022 migraine GWAS clearly pointed to both vascular and central nervous system tissues and cell types. The newly identified loci involve genes encoding known migraine drug targets, namely calcitonin gene-related peptide (CGRP, encoded by *CALCA/CALCB),*) and serotonin 1F receptor (*HTR1F*). The former is the target for CGRP antibodies, and the latter for ditans. In addition, an analysis of about 30,000 patients from the 2022 GWAS with a precise diagnosis of the type of migraine (eg, MO or MwA) showed that three risk variants were specific for MwA (including a SNP in *CACNA1A* the FHM1 gene), two were specific for MO and nine were associated with both types.

Given that some risk loci were found in the Hautakangas 2022 GWAS only, some in the Choquet 2021 GWAS only, and some in both studies, there are now about 180 migraine risk loci.

In addition to these large GWAS including mainly cases with European ancestries, other smaller GWAS conducted in Asia replicated some of the results obtained in European cases and yielded other new SNPs [128]. Another GWAS study conducted in Asian population identified eight novel susceptibility loci correlated with age of migraine onset [129].

Altogether, migraine GWAS have identified more than 180 low-effect-size genetic variants all across the genome, with enrichment in vascular and neuronal cells/tissues, confirming that migraine is a polygenic neurovascular disorder. Recent GWAS demonstrated that all migraine varieties share common molecular mechanisms, and that MO and MwA have specific genetic risk factors and distinct mechanisms. Due to the large size of the samples, clinical data in GWAS were limited and did not permit to study other migraine varieties, such as “pure” MwA (patients having only attacks of MwA and never MO) or chronic MO.

Future studies will have to determine which are the causal genes modified by the SNPs, which are mostly located in non-coding regions, and do not necessarily affect the closest gene. A first challenging step will be to select the list of most-likely causal genes based on GWAS results. For example, the intronic SNP rs9349379 near *PHACTR1* is a proven risk loci for migraine, coronary artery disease, fibromuscular dysplasia, hypertension and cervical artery dissection [130]. A functional study showed that this SNP had no effect on *PHACTR1* but on the gene encoding endothelin-1 (ET-1; EDN1), a strong vascular smooth muscle cells constrictor located 600 kB upstream of the risk SNP [130]. More recent data suggested that SNP rs9349379 may in fact regulate the expression of *PHACTR1*, and not *EDN1*, and that *PHACTR1* could have a role in arterial compliance [131]. This debate on a single SNP shows that enormous amounts of experiments will be necessary to study the 180 migraine SNPs.

In addition, some variants not identified by the mean of GWAS could also be implicated in migraine susceptibility through gene-gene interactions. A case-control study suggested that synergetic effects between a variant in *NRXN2*, coding a component of the synaptic vesicle machinery, and two other genes, *GABRE* and *CASK,* were associated with migraine [132].

### Polygenic risk score and genetic architecture of migraine

The almost 200 variants identified by the latest GWAS each explain only a small fraction of the genetic risk, and their sum do not explain the full heritability of migraine. The Polygenic Risk Score (PRS) assesses the individual genetic risk of migraine as the sum of all SNPs and alleles that increase the risk of migraine carried by an individual. The PRS may be used to analyze the genetic links between the different primary headaches and the different forms of migraine, and between migraine and its comorbidities. The PRS can also assess pharmacogenetic effects [5].

In a study of 1589 migraine sufferers from the Finnish population, familial cases had a significantly higher PRS than non-familial cases [133]. The genetic burden was higher in MwA and FHM compared to MO, and was associated with an earlier age of onset of migraine. These data show that migraine is primarily due to an accumulation of minor variants that produce a favorable (pro-migraine) genetic background, and not to highly deleterious single gene mutations. Noteworthy, a recent study using a PRS derived from 38,872 variants associated with migraine in 8602 subjects in Finland showed a correlation between PRS and migraine diagnoses according to ICHD3 criteria [134]. Non-headache, non-migraine headache, probable migraine, migraine headache, migraine with typical visual aura and hemiplegic migraine formed a continuum along the increasing PRS, which paves the way for the potential concept of genetic classifications [134].

The known small-effect-size pro-migraine variants do not explain the full heritability of migraine. The “missing heritability” may be explained by several hypothesis. First, there are probably hundreds of other small-effect-size variants, which increase the risk of migraine but fall below the required levels of significance in GWAS. Second, persons with multiple disease associated SNPs may have an additive effect conferring a greater overall risk. Third, technical limitations in short read sequencing and Sanger analyses may account for part of this “missing heritability”. Finally, variants with minor or moderate-effect-size probably explain a part of the missing heritability, and cannot be identified by GWAS. A study sequenced the genomic areas associated with migraine in a large cohort of patients and identified four rare variants altering regulatory areas close to four variants discovered by GWAS [135]. Another study analyzed RNA sequencing using a coexpression network of aorta, trigeminal ganglion and visual cortex, combined with a whole sequence genoming. The authors identified a ‘gene module’, a set of coexpressed genes, in the visual cortex that had increased mutations in migraine. Pathway analysis of this module revealed association with hormonal signaling, Alzheimer’s disease, serotonin receptors and heterotrimeric G protein signaling pathway. Noteworthy, mutations in two genes involved in glutamate signalling, *CACNA1B* and *ATXN1*, were found in several migraine families [136]. Using whole-exome sequencing in small populations, new SNPs have been associated with responsiveness to verapamil as a preventive therapy [137], and neurological outcome, including migraine, after head trauma [138]. Finally, “private” large-size-effect variants may be identified by chance, such as in the very rare families carrying mutations of *KCNK18* or *CSNK1D*. In these families, the strong penetrance of the migraine phenotype could result from the cosegregation of the “private” large-size-effect mutation and a pro-migraine genetic background due to a high PRS, whereas most carriers of the same rare variant would not express a migraine phenotype thanks to a non-permissive genetic background. Some authors have even hypothesized that FHM would not be truly autosomal dominant but the result of a rare mutation on a pro-migraine background.

The genetics of migraine thus seems very complex, based on the interaction of hundreds of common small-effect-size variants with rare variants affecting regulatory areas, and with possible “private” moderate to large-effect-size variants.

### Shared genetic background of migraine and its comorbid diseases and traits

**Fig. 3:**
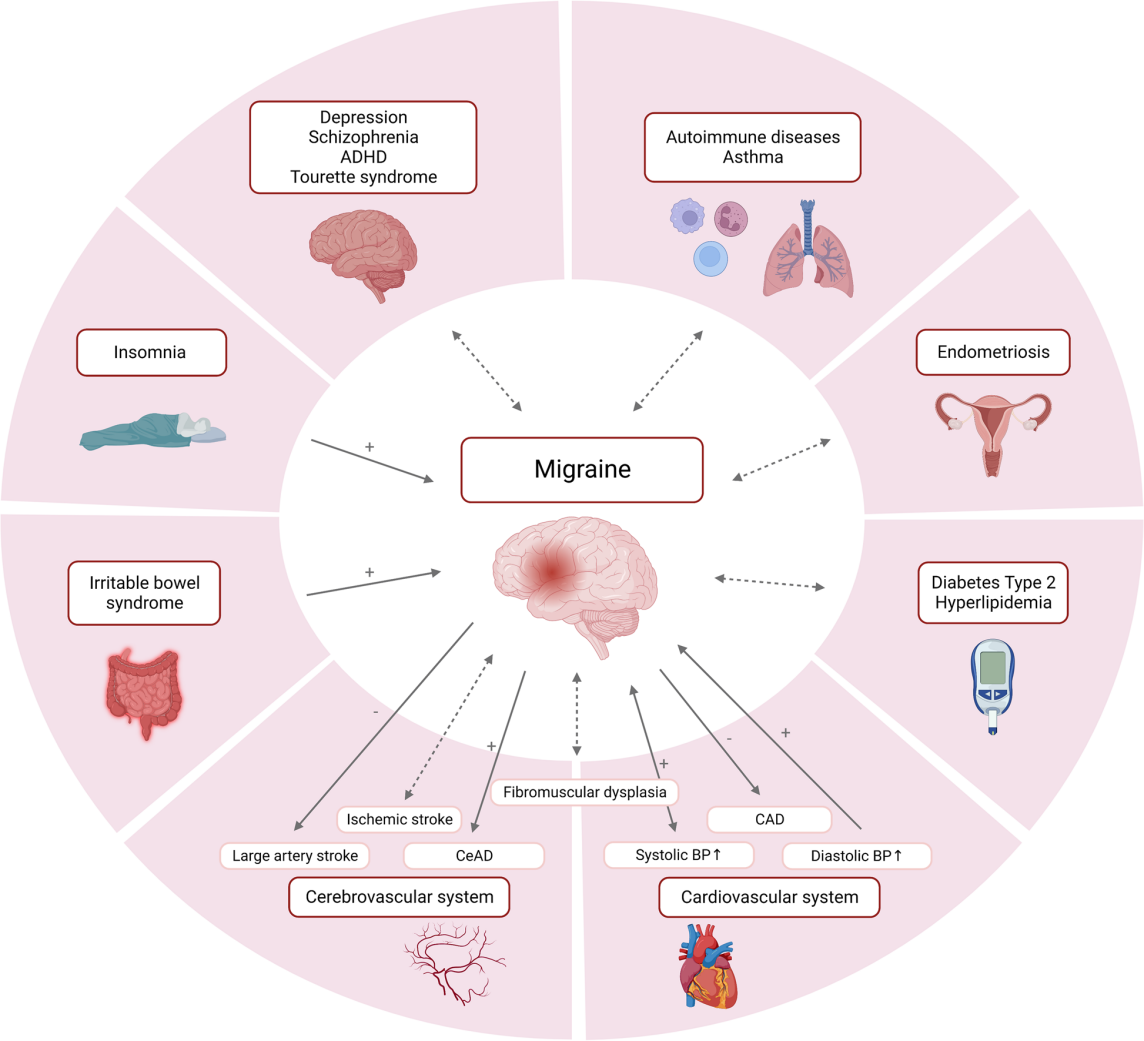
Shared genetic background of migraine and its comorbid diseases. Genetic relation of migraine and some of its clinically most relevant comorbidities. Dotted arrow: Genetic association or correlation as demonstrated by GWAS or genetic correlation studies. Solid arrow: Causal association of genetic variants as demonstrated by Mendelian randomization studies. +, liability to one disease increases risk for the comorbidity; −, liability to one disease decreases risk for the comorbidity; ADHD, attention deficit hyperactivity disorder; BP, blood pressure; CAD, coronary artery disease; CeAD, cervical artery dissection. *Created with BioRender.com*

The comorbidities of migraine are diseases whose prevalence is increased in migraine sufferers compared to controls and for which certain pathological mechanisms could be shared (Fig. 3). Genetics is a tool to explore some of the common mechanisms, firstly by identification of genes associated with both conditions (GWAS), secondly, by estimation of the genetic correlation, namely the proportion of variance the two conditions share due to genetics (genetic correlation studies), and finally, by evaluation of causal relationships between two conditions by using genetic variants as proxies of an exposure (Mendelian randomization [MR] studies) [5, 139].

With regard to genetic studies of associations and correlations, several large studies based on the comparison of GWAS data have shown the existence of a shared genetic susceptibility between migraine and various disorders, including psychiatric disorders [140, 141], ischemic stroke [142], coronary artery disease [143], hypertension [144, 145], sleep disorders [146], and also endometriosis [147], fibromuscular dysplasia [148], type 2 diabetes, hyperlipidemia, autoimmune diseases, asthma, other respiratory conditions [144], restless legs syndrome [149] and hemostatic profile [150]. In contrast, one study found no causal link between genetic susceptibility to migraine and Alzheimer’s disease, intelligence, and brain size [151].

Mendelian randomization studies have provided evidence of causal associations between genetic variants predisposing for migraine and those predisposing for some of the above-mentioned conditions. Findings of Mendelian randomization studies on migraine and comorbid conditions are summarized in Table 4.

**Table 4:** Mendelian randomization studies for causal associations of genetic variants predisposing for migraine and its comorbid disorders and traits

Comorbidity/Trait	Number of patients included		Direction of causality examined	Main finding(s)	Reference
	Migraine	Comorbidity/Trait			
**Cardio−/vascular comorbidities**					
Ischemic Stroke	GERA cohort, UK Biobank and International Headache Genetics Consortium: 85,726 migraine cases and 803,292 controls	MEGASTROKE consortium: 40,585 cases and 406,111 controls	One-directional	No causal associations of migraine and ischemic stroke	Shu et al. [152]
Stroke (ischemic and hemorrhagic)	International Headache Genetics Consortium: Any migraine: 59,674 cases and 316,078 controls MwA: 6332 cases and 144,883 controls MO: 8348 cases and 139,622 controls	MEGASTROKE consortium: All stroke: 40,585 cases and 406,111 controls Ischemic stroke: 34,217 cases and 406,111 controls Hemorrhagic stroke: 3223 cases and 3725 controls	One-directional	No causal association of migraine and stroke, ischemic stroke, and hemorrhagic stroke Migraine liability possibly associated with decreased large-artery atherosclerosis risk	Lee et al. [153]
Stroke and Cervical Artery Dissection	International Headache Genetics Consortium: Any migraine: 59,674 cases and 316,078 controls MwA: 6332 cases and 144,883 controls MO: 8348 cases and 139,622 controls	MEGASTROKE consortium: CeAD: 1393 cases and 14,416 controls All stroke: 40,585 cases and 406,111 controls Ischemic stroke: 34,217 cases and 406,111 controls Large artery stroke: 4373 cases and 297,290 controls Cardioembolic stroke: 7193 cases and 355,68 controls Small vessel stroke: 5386 cases and 343,560 controls	One-directional	Migraine liability associated with increased risk for CeAD Migraine liability associated with decreased risk of large artery stroke	Daghlas et al. [154]
Coronary artery disease and atrial fibrillation	International Headache Genetics Consortium: 59,674 migraine cases and 316,078 controls	Coronary artery disease: 76,014 cases and 264,785 controls Myocardial infarction: 43,676 cases and 128,199 controls Angina: 10,618 cases and 326,065 controls Atrial fibrillation: 60,620 and 970,216	One-directional	Migraine liability associated with decreased risk of coronary artery disease, myocardial infarction, and angina, but not on atrial fibrillation	Daghlas et al. [143]
Blood pressure	International Headache Genetics Consortium: 59,674 cases and 316,078 controls	757,601	Bidirectional	Genetically predicted elevated DBP and SBP, and decreased PP associated with increased migraine risk Migraine liability associated with increased SBP and PP, but not DBP	Guo et al. [145]
Forty-seven traits from the UK Biobank	International Headache Genetics Consortium: 59,674 cases and 316,078 controls	UK Biobank and a combined meta-analysis: for DBP *n* > 1 million individuals	One-directional	Genetically predicted elevated DBP associated with increased migraine risk	Siewert et al. [144]
**Cardiovascular risk factors and lifestyle**					
Alcohol, coffee consumption, smoking	7759 cases and 504,902 controls FinnGen study: 6687 cases and 144,780 controls UK Biobank: 1072 cases and 360,122 controls		Bidirectional	Genetically predicted alcohol and coffee consumption inversely associated with migraine risk, smoking initiation associated with increased migraine risk Migraine liability inversely associated with alcohol consumption, not associated with coffee consumption or smoking initiation	Yuan et al. [155]
Coffee consumption	MwA: 6332 cases and 144,883 controls MO: 8348 cases and 139,622 controls	UK Biobank: 375,833 participants	One-directional	Genetically predicted increase of coffee consumption not associated with the risk of any migraine, MwA or MO	Chen et al. [156]
Smoking	HUNT study: 7522 cases and 35,342 controls		One-directional	No causal relationship between smoking intensity and migraine	Johnsen et al. [157]
**Brain morphometry and cognition**					
Cognition and brain volume	International Headache Genetics Consortium: 59,674 migraine cases and 316,078 controls	Alzheimer’s disease: 71,880 cases and 383,378 controls Measure of general intelligence: 269,867 subjects Measure of intracranial volume: 11,373 subjects Seven subcortical brain volumes ∼ 13,000 subjects	One-directional	Migraine liability not associated with Alzheimer’s disease, intelligence, or any brain volume measures	Daghlas et al. [151]
Brain morphometry	International Headache Genetics Consortium: 59,674 migraine patients vs 316,078 controls	ENIGMA and CHARGE consortia, UK biobank, ABCD cohort: 66,000 participants	Bidirectional	Genetically determined smaller total brain, hippocampal and ventral diencephalon volume possibly associated with increased migraine risk Migraine liability associated with larger volume of the amygdala	Mitchell et al. [158]
**Sleep disorders**					
Insomnia	finn-a-G6_MIGRAINE: 3650 migraine cases and 83,167 controls UK Biobank: 13,597 migraine cases and 449,336 controls	UK Biobank: 462,341 participants	Bidirectional	Genetically determined insomnia significantly associated with increased migraine risk Migraine liability not associated with insomnia risk	Chu et al. [159]
Sleep disturbances	International Headache Genetics Consortium: 59,674 cases and 316,078 controls	UK Biobank: ≥ 237,627 participants	Bidirectional	Difficulty awakening and liability to insomnia symptoms associated with increased risk of migraine Migraine liability slightly associated with increased napping frequency	Daghlas et al. [146]
**Gastroenterological conditions**					
Irritable bowel syndrome	UK Biobank: 1072 cases and 360,122 controls	1121 cases and 360,073 controls	One-directional	Genetically determined irritable bowel syndrome associated with increased migraine risk	Chen et al. [160]
**Gynecological conditions**					
Endometriosis	International Headache Genetics Consortium: 59,674 migraine cases and 316,078 controls	17,054 cases (all stages of endometriosis) and 191,858 controls	One-directional	No causal association	Adewuyi et al. [147]
**Laboratory parameters**					
Clinical chemistry tests (HDL-C, LDL-C, TG, iron, diseases of liver)	29,209 cases and 172,931 controls	Chemistry test values and data on medically relevant disorders from 23,986 to 452,264 participants	One-directional	No causal associations	Tanha et al. [161]
Seventy-nine metabolic traits	International Headache Genetics Consortium: 59,674 migraine cases and 316,078 controls	From 6263 to 24,925	Bidirectional	Genetically predicted shorter length of fatty acids and higher level of a lysophosphatidylethanolamine, LPE(20:4) associated with increased migraine risk	Tanha et al. [162]
Nineteen lipoprotein subfractions	International Headache Genetics Consortium: 54,552 cases and 297,970 controls	47,713 participants	Bidirectional	Lipoprotein subfractions not causally related with migraine	Guo et al. [163]
Serum calcium	International Headache Consortium: 23,285 cases and 95,425 controls	39,400 participants	One-directional	Genetically predicted increase in serum calcium levels associated with increased migraine liability	Yin et al. [164]
Twelve blood-based measures of hemostasis	International Headache Genetics Consortium: 59,674 cases and 316,078 controls	CHARGE Consortium Hemostasis Working Group: *n* = 2583 to *n* = 120,246	Bidirectional	Increased FVIII, vWF, and phosphorylated fibrinopeptide A and decreased fibrinogen associated with increased migraine risk (especially MA) Migraine liability not associated with measures of hemostasis	Guo et al. [150]
Vitamine D Levels	GWAS 1 (Hautakangas et al.): 48,975 cases and 540,381 controls GWAS 2 (Choquet et al.): 28,852 cases and 525,717 controls GWAS 3 (FinnGen): 10,536 cases and 208,845 controls	UK Biobank 417,580 participants	Bidirectional	Genetically determined increase in circulating vitamin D levels associated with a decreased migraine risk Migraine not associated with vitamin D levels	Niu et al. [165]
Circulating Insulin-Like Growth Factor 1 Levels	GWAS 1 (FinnGen): 10,536 cases and 208,845 controls GWAS 2 (Choquet et al.): 28,852 cases and 554,569 controls	UK Biobank 363,228 participants	Bidirectional	Genetically determined increase in IGF1 levels associated with decreased risk of migraine Migraine not associated with IGF1 levels	Abuduxukuer et al. [166]
DNA methylation	International Headache Genetics Consortium: 59,674 cases and 316,078 controls	DNA methylation quantitative trait loci from whole blood, *n* = 639 and fetal brain, *n* = 166	One-directional	Not provided	Hannon et al. [167]
**Socioeconomic outcomes**					
School absence and educational attainment	Avon Longitudinal Study of Parents and Children: 6113 participants		One-directional	Migraine liability not associated with school absence or educational attainment in childhood and adolescence	Hughes et al. [168]
Nineteen social and socioeconomic outcomes	UK Biobank: 10,603 cases, 326,394 controls		One-directional	Migraine liability associated with reduced chance of having a weekly leisure or social activity	Harrison et al. [169]

## Other recent genetic findings

### Genetics of headache

A 2018 British GWAS studied 74,461 individuals who had had a headache interfering with daily activities in the previous month and 149,312 controls [170]. The majority of patients probably had tension headache and less often migraine. This study identified 28 headache susceptibility loci of which 14 had already been identified by GWAS in migraine, and 14 were new. The majority of the potential headache genes were neuronal and not vascular. This study also found a shared genetic background between the headache phenotype and many psychological traits associated with vulnerability to depression and negative emotions, highlighting the importance of links between psychiatric conditions and painful conditions.

Another recent GWAS including 2084 Taiwanese patients and 11,822 age- and sex-matched controls identified two loci, rs10493859 in *TGFBR3* and rs13312779 in *FGF23*, both functionally relevant to vascular function and migraine, to be significantly associated with self-reported headache [171].

Until recently, studies on genetics of Cluster headache (CH) have been dominated by candidate gene studies with conflicting findings [172, 173]. A first Italian GWAS on 99 patients and 360 controls identified *ADCYAP1R1* and *MME* gene variants as possibly associated with susceptibility for CH [174]. These findings were not replicated in a larger Swedish cohort [175]. In 2021, two GWAS out of which one included Dutch cases (*n* = 840) and controls (*n* = 1457) and Norwegian cases (*n* = 144) and controls (*n* = 1800) [176], and the second one UK cases (*n* = 852) and controls (*n* = 5614) and Swedish cases (*n* = 591) and controls (*n* = 1134) [177] independently identified four risk loci for CH on chromosome 1, chromosome 2 (two loci), and chromosome 6, respectively. Subsequently, a meta-analysis of both studies analyzing 8,039,373 variants confirmed a significant association of all 8 index variants (in the 4 loci) and identified three additional loci with genome-wide significance on chromosomes 7, 10 and 19. The nearest genes to the loci on chromosome 2 and 6 are *MERTK* and *UFL1/FHL5*, respectively. Interestingly, as stated in Table 3, *UFL1/FHL5* has previously been identified as a migraine risk locus [120].

### Genetics of chronic migraine

Chronic migraine is the most disabling form of migraine, and causes for migraine chronification remain incompletely understood. In order to identify genetic variants contributing to migraine chronification, a comparison of patients with chronic migraine and patients with episodic migraine is necessary, whereas most studies attempting to find genetic risk factors have compared chronic migraineurs to healthy controls. Recent studies comparing chronic and episodic migraine have found genetic variants in the *TRPM8* gene [178], the *TRPV1* gene [179], and HLA class I alleles [180] to be associated with chronic migraine.

Previous to these studies, a candidate gene-association study examined 144 SNPs from 48 candidate genes in patients with chronic or high-frequency episodic migraine compared to healthy controls, and did not reveal any significant findings [181].

The first study assessing whole-genome sequencing data in patients with chronic compared to episodic migraine did not show any significant difference [182].

Further studies are needed to determine the proportion of genetic and environmental factors in chronic migraine.

### Genetics factors underlying treatment response

Genetic factors strongly influence the absorption, distribution, metabolism and excretion of drugs. Studies addressing genetic factors underlying treatment response to triptans have described *GNB3 C825T* gene polymorphism to be associated with a better response to triptans in CH patients, and polymorphisms in the *PRDM16, SLC6A4* and *DRD2* genes to be associated with a better, inconsistent and worse response to triptans in migraine patients, respectively [183]. Another study showed that a polygenic risk score doubling the risk of migraine was associated with a better response to triptans [184].

Recently, whole-exome sequencing in a discovery cohort of migraine patients treated by verapamil (definitive responders *n* = 21 and definitive non-responders n = 14), followed by genotyping in a confirmation cohort (*n* = 185), identified 13 SNP, which were highly correlated with the changes in the number of migraine days [137].

In the future, determining the genetic profile of an individual could allow the choice of treatments with the best profile of efficacy and tolerance [5].

### Mitochondrial DNA and migraine

Mitochondrial dysfunction has been suspected to contribute to migraine pathophysiology, since migraine-like headache is a clinical feature of several mitochondrial diseases [185–187], presumable mitochondrial biomarkers have been found to be elevated in migraine patients [188], and several studies have reported mitochondrial DNA (mtDNA) candidate variants possibly associated with migraine [189, 190]. However, the first GWAS assessing 775 mitochondrial DNA variants in 4021 migraine sufferers and 14,288 controls found no migraine-associated variants, ruling out the mitochondrial hypothesis suggested by older studies [191]. Limitations discussed by the authors were the diagnosis of migraine based on a questionnaire covering symptoms during the past 12 months instead of a clinical interview, and the absence of consideration of heteroplasmic variation, copy-number variations and epigenetic changes.

## Conclusion

Genetics of migraine have made significant progress over the past 15 years [5, 28, 114]. The study of monogenic migraines identified key proteins of the susceptibility to CSD and helped to better appreciate the links between migraine and vascular disorders. GWAS have identified multiple susceptibility genes revealing several complex networks of “pro-migraine” molecular abnormalities, mainly neuronal and vascular (Fig. 1). Genetics has also underscored the importance of genetic factors shared between migraine and its major co-morbidities including depression and high blood pressure. Very large-scale studies are still needed to map all of the susceptibility loci to migraine and then to understand how these genomic variants lead to migraine cell phenotypes. Ultimately, the main pathophysiological mechanism in a given patient, neuronal or vascular or otherwise, could be determined through its genetic risk profile. Pharmacogenetics could help predict the therapeutic response and thus help prescribe the treatment with the best safety and efficacy profile.
